# Variable Responses to Corneal Grafts: Insights from Immunology and Systems Biology

**DOI:** 10.3390/jcm9020586

**Published:** 2020-02-21

**Authors:** Antonio Di Zazzo, Sang-Mok Lee, Jaemyoung Sung, Matteo Niutta, Marco Coassin, Alireza Mashaghi, Takenori Inomata

**Affiliations:** 1 Ophthalmology Complex Operative Unit, Campus Bio Medico University, 00128 Rome, Italy; antoniodizazzo@gmail.com (A.D.Z.); matteoniutta@libero.it (M.N.); m.coassin@unicampus.it (M.C.); 2 Department of Ophthalmology, Catholic Kwandong University College of Medicine, Gangneung-si, Gangwon-do 25601, Korea; lsm10003@gmail.com; 3 Department of Cornea, External Disease & Refractive Surgery, HanGil Eye Hospital, Incheon 21388, Korea; 4 University of South Florida, Morsani College of Medicine, Tampa, FL 33612, USA; jsung1@usf.edu; 5 Department of Ophthalmology, Juntendo University Faculty of Medicine, Tokyo 1130033, Japan; 6 Systems Biomedicine and Pharmacology Division, Leiden Academic Centre for Drug Research, Leiden University, 2333CC Leiden, The Netherlands; 7 Department of Strategic Operating Room Management and Improvement, Juntendo University Faculty of Medicine, Tokyo 1130033, Japan; 8 Department of Hospital Administration, Juntendo University Faculty of Medicine, Tokyo 1130033, Japan

**Keywords:** cornea, transplantation, immune rejection, genomics, biomechanics, personalized medicine

## Abstract

Corneal grafts interact with their hosts via complex immunobiological processes that sometimes lead to graft failure. Prediction of graft failure is often a tedious task due to the genetic and nongenetic heterogeneity of patients. As in other areas of medicine, a reliable prediction method would impact therapeutic decision-making in corneal transplantation. Valuable insights into the clinically observed heterogeneity of host responses to corneal grafts have emerged from multidisciplinary approaches, including genomics analyses, mechanical studies, immunobiology, and theoretical modeling. Here, we review the emerging concepts, tools, and new biomarkers that may allow for the prediction of graft survival.

## 1. Introduction

The cornea is the most frequently transplanted human tissue [1]; however, 10% of recipients with uninflamed graft beds will experience graft failure. Despite maximal immune suppression, the failure rate dramatically increases to 50% in recipients with inflamed graft beds. The likelihood of acute rejection and/or graft failure in patients in this “high risk” category is comparable to or larger than that of the commonly transplanted solid organs [2]. Despite intensive study, the heterogeneity of responses to corneal grafts is a poorly understood subject. Interdisciplinary approaches have been increasingly applied to resolve this heterogeneity and to find new biomarkers for disease prediction. Novel genetic, immunological, and physical biomarkers have recently been introduced. Here, we review the recent developments in the field with a special focus on new innovations and emerging frontiers.

## 2. Emerging Immunological Markers

With the high frequency of corneal transplants, numerous studies have attempted to decode the overarching etiologies of corneal allograft rejection. As with other solid organ transplants, the immune system has been shown to be one of the most important contributors to corneal allograft rejection, along with immune-enabling factors such as blood and lymph flow, in the relatively immune-tolerant cornea. Major and minor histocompatibility complexes, immune cell milieu, native corneal cell dynamics, and proteins such as cytokines and cell surface proteins have been investigated for their contributions to allograft rejection. Although efforts to understand the pathophysiology of corneal allograft rejection continue, here we discuss contributory factors already identified by this field of study (Table 1).

### 2.1. Importance of Immune Processes in Cornea Transplantation

**Figure 1:**
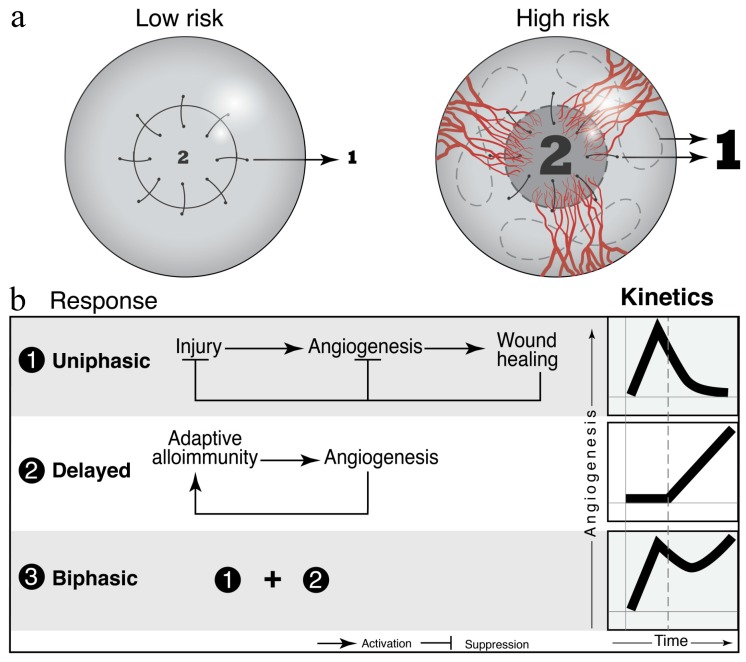
Vascular dynamics in the grafted cornea and in the host bed as predictive and prognostic biomarkers for graft survival in murine models. (**a**) The density of pre-existing vessels correlates with the risk of allograft rejection. “Low risk” and “high risk” murine models are commonly used to study the host response to grafted corneas. (**b**) Wound healing and adaptive immune processes contribute to the angiogenic response to cornea grafts. Figure taken from Azimzade, Y. et al. with permission [43].

Although corneal graft rejection involves a complex pathophysiology, the host immune response seems to be the most important modulator. Specifically, the transfer of donor immune cells and the strong cluster of differentiation (CD)4+ T helper (Th)1 cell-mediated delayed-type hypersensitivity reactions brought on by allogeneic components of the donor cornea are thought to be the main culprits [3,42]. The anterior chamber of the eye is one of the few organs that carries “immune privilege” This privilege is partially due to the lack of draining lymphatic vessels and to a unique interplay between immunomodulatory molecules that suppress angiogenesis [17,18,19,20]. However, the neovascularization and lymphangiogenesis initiated by ocular inflammation post-operation allows for the transfer of donor antigens to draining cervical lymph nodes (Figure 1). The subsequent recognition of donor antigens as foreign molecules leads to the activation and proliferation of naïve T cells to effector T cells (Teff) that trigger the destruction of graft cells [42].

Although immunosuppressive therapies, including steroids, cyclosporin, and tacrolimus, have demonstrated benefits in preventing acute graft rejection, the risk of corneal graft rejection remains as high as 40–90% in high-risk host beds. Infections at the surgical site, complications due to existing autoimmune diseases, and angio/lymphangiogenesis from previous grafts are detrimental to graft outcomes and increase the risk of long-term side effects of immunosuppressive medications, including drug toxicity, glaucoma, and cataracts [44,45,46,47]. Additionally, the severity of side effects associated with immunosuppressants has led to disagreements regarding the timing of when therapy should be discontinued. Therefore, the identification of graft rejection biomarkers is imperative for the development of preventative and precision pharmacotherapies to avoid rejection and to help determine effective immunosuppressant dosing in cases where corneal graft rejection does occur (Table 1). In the following sections, we explore the current understanding of anatomical changes in the cornea post-transplant and the cellular and molecular interactions between the aforementioned immune cells, antigen-presenting cells (APCs), cytokines, and major histocompatibility complexes (MHC).

### 2.2. In Vivo Confocal Microscopy Evaluation of Immune Cells in the Corneal Graft

Slit lamp biomicroscopy has been the gold standard for examining clinical signs of corneal allograft rejection. However, research has indicated that the early detection of immune responses is crucial for the prompt diagnosis of graft rejection, and this limits the use of slit lamp biomicroscopy. Specifically, conventional light microscopy produces light that is reflected from structures surrounding the focal area. The resultant fringing effect leads to images that are low-contrast and ultimately of lower resolution [48]. The resolution power of slit lamp biomicroscopy is often constrained at 20 µm. Therefore, the visualization of immune-mediated signs of graft rejection, such as delayed-type hypersensitivity reactions against allogeneic cells and leukocytic infiltrations at the graft site, are limited [9]. These limitations may lead to a failure to recognize early signs of graft rejection, a delay in the diagnosis of graft rejection, and irreversible chronic damage.

In vivo confocal microscopy (IVCM) is now utilized to overcome these limitations. IVCM also overcomes some additional limitations of conventional light and electron microscopy, including eliminating the requirements for an in vitro study environment and for the fixation processes that chemically and physically disrupt the cells taken in vivo. By isolating a single focal plane and obscuring the planes that are not in focus, IVCM improves both the lateral and axial resolution to 1–2 µm and 5–10 µm, respectively, and allows for the visualization of leukocytic infiltrates that can be as small as 7 µm [48]. This level of resolution—in the context of corneal layer examinations—allows for the visualization of additional minute microstructural changes to keratocytes and cuboidal cells in the basal epithelium, corneal stroma, and endothelium.

Previously, Chirapapaisan C et al. reported that there was a significant increase in immune cell density following corneal graft rejection, along with a correlation between sub-basal layer immune cell density and clinical signs of graft rejection [10]. Of note, the density of immune cells was significantly increased in the sub-basal and endothelial layers in rejected grafts compared with that in nonrejected grafts. A measurement of seven nonrejected grafts at one year post-operation showed that there was a significant decrease in immune cells in all layers. This finding suggests that there are long-term differences in immune cell dynamics between rejected and nonrejected grafts. A history of dry eye disease in the donor has also been shown to contribute to the rejection of corneal allografts in a mouse model. It is possible that increased leukocyte maturation and T-cell dysregulation triggered by dry eye disease may participate in the pathogenesis of allograft rejection [16]. Recently, studies have reported that atypical hyper-reflective cells and activated keratocytes in the corneal stroma are crucial biomarkers of corneal allograft rejection [10,11,16].

As quiescent keratocytes remain dormant in their native state, activated keratocytes appear to reflect the level of intrastromal inflammation in response to cytokines and other inflammatory mediators, including interleukin (IL)-1 and tumor necrosis factor (TNF)-α [13,14,23,25]. Moreover, Beauregard et al. [17] reported that an inflammation-independent alloimmune response caused by delayed-type hypersensitivity reactions leads to apoptosis of keratocytes in the stroma and contributes to graft failure [12]. The increased level of activated keratocytes can be observed as early as two months before clinical diagnosis of corneal allograft rejection, and reaches its peak at the time of diagnosis. The subsequent administration of topical steroids and/or cyclosporin at diagnosis has been shown to successfully decrease activated keratocyte levels within one month of therapy [9]. Therefore, the current understanding of corneal allograft rejection pathogenesis dictates that corneal immune cell infiltration may be a decisive early biomarker. Thus, new or increased immune suppressant therapy should be considered following IVCM examinations that demonstrate increases in this immune cell population.

### 2.3. Cytokines and Regulatory T cells

Other components of the immune milieu, including various cytokines and mediator cells, contribute to the decompensation of the corneal endothelium when their balance shifts [42,49]. Corneal edema and bullous keratopathy often signal graft failure, and these conditions are often characterized by dystrophic changes in the Descemet’s membrane and stroma that are partially, if not largely, caused by immune responses [50]. Keratocytes within the epithelium and stroma, endothelial cells, and immune cells brought to the cornea through homing mechanisms all contribute to the corneal immune response through cytokine and chemokine secretion, assisted by the expression of various adhesion proteins in the corneal cells [21,24,26,28,29,30,34,35,36,37]. Very similar immune components have been identified in the anterior chamber after penetrating keratoplasty, suggesting that graft rejections are likely initiated within the corneal microcosm and that identifying minor dysregulations holds the key to timely detection of allograft rejection.

Yoon et al. proposed that CD8^+^/interferon (IFN)-γ^+^ cell and C3a levels within the aqueous humor (AqH) at weeks 2–4 post-keratoplasty may be predictors of graft rejection. This proposition was supported by the results of a receiver operator characteristic (ROC) curve analysis with an area under the curve (AUC) of 0.715 and 0.847, respectively [32]. Although this analysis was from a retrospective study that used a xenotransplantation model, the high reported specificity of 0.94 and 1.0, respectively, at the optimal cutoff warrants further investigation. Yamauchi et al. identified numerous candidate biomarkers that were correlated with the development of bullous keratopathy and low endothelial cell density, including IL-1a, IL-8, IL-17A, TNF-a, granulocyte-macrophage colony-stimulating factor (GM-CSF), macrophage inflammatory protein (MIP)-1a, IFN-c, and E-selectin levels in the AqH [31]. Intriguingly, Maier et al. investigated the risk of rejection before corneal transplantation and reported that the levels of protective factors (IL-2 and IL-5) and hazardous factors (IL-4 and IFN-γ) within the AqH before operation aided in prognosticating postoperative immune responses [27].

Regulatory T cells (Treg) were first identified in 1995 and are a major subgroup of T cells of paramount importance in the development of self-tolerance and downregulation of immune responses [51]. With respect to corneal allografts, IL-10 and TGF-β secreted by Foxp3^high^-expressing Tregs found in the draining lymph nodes of allograft recipients have been associated with reduced graft rejection [52]. Similarly, Foxp3 expression in T cells is reduced in recipients who experience corneal allograft rejection. These findings highlight the role of Tregs in the prevention of corneal graft rejection. Unfortunately, the differentiation and proliferation pathways of Tregs are very susceptible to changes in the environment, with fluctuations altering the immunosuppressive capabilities of these cells. Previous studies have demonstrated that high-risk host beds, often characterized by inflammation, neovascularization, and lymphangiogenesis, are correlated with a stark decrease in the functionality and expression of Foxp3 in Tregs in draining lymph nodes after corneal allograft placement [16,40]. Similarly, studies evaluated the use of Foxp3 expression level as a biomarker for kidney allograft prognosis and reported that there is a positive correlation between increased Foxp3 expression at 6 months post-transplantation and increased kidney function at 2 years post-transplantation [41]. It is possible that localization and expression of Foxp3^+^ Tregs—in concert with the various aforementioned cytokines and chemokines—could be potent predictive biomarkers for corneal graft rejection.

### 2.4. Major Histocompatibility Complex and Antigen-Presenting Cells

A healthy cornea exhibits a unique distribution of dendritic cells. MHC class I-positive dendritic cells are present throughout its entirety, while MHC class II-positive cells only reside in the limbus and peripheral cornea [53,54]. Despite the distinct separation in occupying regions, both classes of MHC are thought to contribute to the development of graft rejection [42]. This hypothesis is further underscored by the increased expression of Class II MHCs in the resident corneal dendritic cells and epithelial Langerhans cells within the central cornea during states of inflammation, including those caused by corneal transplantation [7,38,39,55].

Yet, unlike in kidney and heart transplants, human leukocyte antigen (HLA) matching is not routinely performed in corneal transplantation because the largest randomized studies that compared the outcomes of HLA matching (i.e., the Collaborative Corneal Transplantation Studies (CCTS)) reported that there were no significant differences in graft rejection rates [3]. Although the absence of meaningful extenders of corneal graft survival time and the predetermined notion that the cornea is immune-privileged have pushed clinical practice away from the utilization of HLA matching, continued conflicting reports have flooded the field and fueled further research [5,6]. New observations of increased Class II MHCs and co-stimulatory molecules, such as CD40, CD80, and CD86, in grafts from high-risk beds highlight the importance of MHC discrepancies in recipients who are at a high risk for graft rejection [4,8].

MHC class I-related chain A (MICA) has also been identified as a potential biomarker of graft rejection [33]. MICA is a protein that is expressed in the cytoplasms of corneal epithelial and endothelial cells; however, there is not much known regarding its regulatory mechanisms or impact on allograft rejection. Nevertheless, IFN-γ, a key cytokine released by CD4+ Th1 cells, has been shown to cause an upregulation of MICA in the epithelium, and this upregulation subsequently stimulates CD8+ T cells and natural killer cells [33]. This link between increased IFN-γ levels observed in rejected grafts and the enhanced immune response against the corneal epithelium suggests that changes in MICA levels may be used to preemptively recognize epithelial damage caused by graft rejection.

Dendritic cells (DCs) are bone marrow-derived APCs that are capable of expressing both MHC class I and II molecules; thus, they are thought to play an important role in the pathogenesis of graft rejection. Recent studies have alluded to the role of DCs as biomarkers for corneal epithelial inflammation in patients with type 2 diabetes mellitus, suggesting they may also be used as biomarkers for graft-related inflammation [56]. Nuanced variations in the presence, phenotype, and maturity of APC and DC populations have been shown to have varying associations on corneal allograft transplantation outcomes, implying they may reveal underlying immune dynamics [16,57,58]. Hamrah et al. reported that the number and distribution of mature CD11c+ DC populations is increased in the anterior stroma and that there is an upregulation of MHC class II molecules within 24 h after inflammation is induced. Additionally, CD11c-CD11b+ monocytes/macrophages, which are normally confined to the posterior stroma, have been found throughout the stroma during inflammation [38]. APCs, including corneal DCs, monocytes, and macrophages, are the cornerstone of immune responses and reflect the immune state; therefore, future studies should investigate the role of these cells in corneal graft rejection.

## 3. Endothelial Cell Density and Morphological Indicators as Graft Response Predictors

Human corneal endothelial cells (HCECs) cover the posterior surface of the cornea on Descemet’s membrane in a single layer with a well-arranged mosaic pattern. They play an important role in keeping the cornea clear by pumping out water into the anterior chamber using Na^+^-and K^+^-dependent ATPase in the basolateral membrane [15]. Unlike the vascular endothelium that originates from the mesoderm, HCECs originate from the cranial neural crest (neuroectoderm) [59,60]. HCECs have a limited proliferative capacity in vivo, and damage to HCECs by trauma; surgery; or primary corneal endotheliopathies, such as Fuchs’ corneal endothelial dystrophy, can lead to bullous keratopathy or corneal endothelial blindness. Corneal endothelial dysfunction is the main cause for corneal transplantation, responsible for 40% to 50% of transplants [61,62]. Endothelial cell density (ECD, cell counts/mm^2^) and endothelial cell morphology (hexagonality and coefficient of variation of mean cell area) are markers for corneal endothelial dysfunction [15,63]. The first direct visualization of the endothelium by Vogt was performed using the specular reflection method with the slit lamp in 1918, and the specular microscope was developed by David Maurice in the 1960s to evaluate ECD and morphology for ophthalmic research [64]. The specular microscope is now widely used clinically to evaluate corneal endothelial function in diverse corneal disease conditions and in the preoperative evaluation for intraocular surgery. For corneal transplantation, minimum donor ECD has been generally established to be 2000 cells/mm^2^ for penetrating keratoplasty. In a longitudinal cohort study of 500 consecutive penetrating keratoplasties, a low endothelial cell density before surgery (*p* = 0.007) and 2 months postoperatively (*p* = 0.002) were identified as significant risk factors for developing late endothelial failure [65]. However, a recent study conducted by the Corneal Donor Study Investigator Group revealed that graft failure from endothelial decompensation was not related to donor ECD; nevertheless, they reported that graft failure was strongly correlated with ECD at 6 months after penetrating keratoplasty [66,67]. Among endothelial cell morphology indices, only lower hexagonality at 6 months after penetrating keratoplasty showed a suggestive trend of higher graft failure (*p* = 0.02) [67]. Recently, newer surgical techniques for endothelial dysfunction, including Descemet’s stripping automated endothelial keratoplasty (DSAEK), Descemet’s membrane endothelial keratoplasty (DMEK), and Pre-Descemet’s endothelial keratoplasty (PDEK) have been used to replace the standard technique of penetrating keratoplasty [68]. Studies have reported that cell loss is greater in the first six months after endothelial keratoplasty than in the first six months after penetrating keratoplasty; therefore, the minimum donor ECD requirement is 2300–2500 cells/mm^2^) [69]. In a recent study evaluating the factors associated with graft survival and ECD after DSAEK, lower graft ECD was identified as a significant predisposing factor for lower postoperative ECD, but was not a predisposing factor for graft failure [70]. For DMEK, lower graft ECD was also found as a significant risk factor for higher postoperative ECD loss by multinominal regression analysis comparing groups of eyes with low and high endothelial cell loss [71]. In a genome-wide association study of specular microscopic findings in 6125 Icelanders, an intergenic variant (rs78658973(A), frequency = 28.3%) close to ANAPC1 (anaphase-promoting complex subunit 1) was strongly associated with decreased ECD [72]. ANAPC1 encodes a cell cycle-regulated E3 ubiquitin ligase that controls the progression through mitosis and the G1 phase of the cell cycle. Sequence variation at ANAPC1 accounts for 24% of the variability in corneal ECD [72].

Transplantation of cultured HCECs or possible precursor cells has been performed to overcome the shortage of donor tissue [59,73,74,75,76]. Diverse research groups have identified markers for HCECs, including CD166, glypican 4 (GPC4), CD200, CD56, Integrin Subunit Alpha 3 (ITGA3), and CD49c [77,78,79,80,81]. To discriminate HCECs from other cell types, molecular markers have been evaluated by integrating the published ribonucleic acid (RNA)-seq data of corneal endothelial cells (CECs) with the FANTOM5 atlas, which contains a diverse range of cell types. CLRN1, MRGPRX3, HTR1D, GRIP1, and ZP4 were identified as markers of CECs [82]. Recently, Kinoshita et al. reported promising clinical results by injecting cultured HCECs supplemented with a rho-associated protein kinase inhibitor into the anterior chamber [76]. To assess the quality of in vitro cultured HCECs, surface markers were analyzed using flow cytometry, and CD166+/CD24–/CD105–/CD44– cells were defined as effector cells in this group [61]. However, to measure the quality of cultured HCECs noninvasively, they developed the “spring constant K” as a physical biomarker, which represents the collective order of HCECs and is calculated by the second derivative of the function summated for the number of neighbor cells according to the distance from each reference cell [61]. The quantitative analysis of spring constant K from the effective interaction potential can be used preoperatively in vitro using phase contrast microscopy images and postoperatively in vivo using specular microscopy images. While preoperative spring constant K showed a clear positive correlation with effector cell fraction (*r*^2^ = 0.86) in vitro, its best classification accuracy (AUC = 0.96) was found with postoperative ECD in vivo at 6 months in comparison with other parameters, including effector cell fraction, preoperative ECD, and preoperative hexagonality [61]. This biomarker may enable preemptive interventions from passive monitoring in patients with severe corneal disorders [61]. The biomarkers for graft response covered in this section are summarized in Table 2.

## 4. Vascular Dynamics and Graft Survival

### 4.1. Prior to Transplantation

The normal cornea is devoid of blood and lymphatic vessels and actively maintains a state of “angiogenic privilege” [83]. Corneal grafting onto vascularized and inflamed host beds (i.e., high-risk beds) leads to increased angiogenesis that further increases the chance of graft rejection [57]. Thereafter, the usually high success rate of corneal transplantation is completely overshadowed by rejection of larger grafts. Host bed vascularity is the principal risk factor for allograft rejection because corneal neo-vessels are critical in delivering the immune effector cells to the graft site and in driving the immune rejection [84].

The likelihood of the birth of a new vessel branch at any location is suggested to be proportional to the concentration of angiogenic factors and inversely proportional to the distance to the source. When angiogenesis develops with limited reversibility within a short time scale, prompt assessments of corneal neovascularization biomarkers in the early stage are critical for adequate and successful management. Corneal angiogenesis can occur from excessive levels of pro-angiogenic factors, including vascular endothelial growth factor (VEGF), basic fibroblast growth factor (bFGF), matrix metalloproteinases (MMPs), and others [22,85,86,87,88,89]. The main markers implicated in angiogenesis and lymphangiogenesis are listed in Table 3. Members of the VEGF family (VEGF A, C, and D) are directly involved in both corneal angiogenesis and lymphangiogenesis [22,85,89,90,91]. Corneal angiogenesis can also occur secondary to a relative paucity of anti-angiogenic factors [92,93], such as soluble VEGF receptors (sVEGFR)-1, -2, -3; pigment epithelium-derived factor; angiostatin (created by the proteolytic cleavage of plasminogen); and endostatin (a type XVIII collagen proteolytic product) [94,95,96,97]. In response to hypoxia, the expression of VEGF increases 30-fold within minutes. Additionally, studies have reported that VEGF-A is directly involved in the pro-angiogenic circle of “bad” neovascularization, which allows a direct immune response to the graft site. This neovascularization ultimately induces allograft reactions and rejections [83]. Conversely, studies have revealed that IFN-γ has high antiangiogenic activity, though it is a powerful promoter of T cell-mediated immune rejection [83].

Several reports underline the critical proangiogenic actions of IL-17 and IL-1a, local biomarkers of corneal angiogenic response to insults such as grafting. Importantly, the survival of corneal transplants can be significantly affected by the degree of the hemangiogenic, as well as lymphangiogenic, response [83]. This finding is related to lymphatic and blood vessels acting as mediators of the afferent and efferent arms of the immune system, respectively. Lymphatics mediate the trafficking of alloantigen and immune cells from the graft to lymphoid tissues, and blood vessels facilitate the homing of immune cells to the graft [83]. Both angiogenesis and lymphangiogenesis are mainly driven by the VEGF family of receptors and ligands. The binding of VEGF-A and VEGF-B to VEGFR-1 and VEGFR-2 drives angiogenesis, whereas the binding of VEGF-C and VEGF-D to VEGFR-2 and VEGFR-3 drives lymphangiogenesis [91,98,99,100]. The complex interactions between VEGFs and VEGFRs exhibit sophisticated control over angiogenesis and lymphangiogenesis [101].

In addition, macrophages (MPs), a subtype of innate immune cells, contribute to proliferation of lymphatic vessels in inflammation-associated lymphangiogenesis [102,103]. Corneal lymphangiogenesis has been shown to be associated with CD11b^+^ MPs [103], and under inflammatory conditions, CD11b^+^ MPs secrete VEGF-A and VEGF-C in the peripheral cornea (limbus) [104]. The presence of lymphatic vessels in the cornea is usually detected by positive localization of podoplanin, lymphatic vessel endothelial hyaluronan receptor 1 (LYVE-1), VEGF-C, or VEGFR-3 and is associated with blood vessels, inflammatory cells, and disorganized stromal architecture in the immediate vicinity. Hence, VEGFR-3 is a specific biomarker for lymphangiogenesis. Additionally, the central corneal presence of CD11b^+^ macrophages may also play a direct role in corneal lymphangiogenesis.

Finally, studies have also suggested that very late antigen 1 (VLA-1) correlates with allogeneic corneal transplant survival. VLA-1 is an integrin that mediates intercellular and matrix interactions, and VLA-1 blockade markedly promotes survival of corneal allografts [102]. Chen L, et al. has been implicated in inflammatory reactions involving hemangiogenesis, macrophages, and T cells. Recently, VLA-1 has been identified in the corneal stroma.

**Table 3:** Markers of angiogenesis and lymphangiogenesis.

Antiangiogenic	Proangiogenic
IFN-γ [83]	VEGF-A, C, D [22,85,89,90,91,98,99,100]
sVEGFR-1,2,3 [92,93,94,95,96,97,98,99,100]	bFGF [22,85,86,87,88,89]
PEDF [94,95,96,97]	VLA-1 [102]
Endostatin [94,95,96,97]	PDGF [105,106]
	ANG2 [107,108]

Abbreviation listed as followed. IFN-γ: interferon gamma; sVEGFR: soluble VEGF receptors; PEDF: pigment epithelium-derived factor; VEGF: vascular endothelial growth factor; bFGF: basic fibroblast growth factor; VLA-1: very late antigen 1; PDGF: platelet-derived growth factor; ANG2: angiopoietin 2.

### 4.2. After Transplantation

Previous studies that focused on post-transplantation angiogenic responses revealed that low-risk (LR) and high-risk (HR) hosts have comparable lymphatic ingrowth early on; however, the amount of blood vessel ingrowth is significantly different [83]. These data demonstrate that angiogenic homeostatic mechanisms are restored much earlier in LR as opposed to HR graft recipients. Specifically, lymphatic responses regress early in noninflamed LR hosts, and this regression critically reduces the risk of allograft rejection. Corneal neovascularization is established in vitro by CD31 immunoreactive profiles. Simultaneous immunofluorescence using CD31 and podoplanin distinguishes lymphatic vessels from blood vessels. Moreover, confirming the presence of lymphatic mRNA by identifying at least two of the following mRNAs: podoplanin, VEGFR-3, and LYVE-1, may improve the accuracy of detection post-transplantation [109].

Different markers, including podoplanin, LYVE-1, and VEGFR-3, are commonly used to identify lymphatic channels. CD31 is a traditional marker of blood vessels that is weakly expressed on lymphatics [110]. However, podoplanin is a more specific marker of lymphatic endothelium since it is absent from blood vessel endothelium [111]. Conversely, LYVE-1 has been found to be expressed in some macrophages and blood vessels, including liver sinusoids [110]. Similarly, VEGFR-3 is found in some blood vessels, including in cases of inflammation-induced angiogenesis [110]. However, a recent “Consensus Statement on the Immunohistochemical Detection of Ocular Lymphatic Vessels” suggests that the use of a single lymphatic marker is sufficient for areas in which the presence of lymphatics has been well established, including the inflamed cornea [112].

In human beings and mice, angiopoietin 2 (ANG2) is mainly expressed in the epithelium and mildly expressed in the endothelium of the avascular cornea. It is, however, expressed in the epithelium, endothelium, and stroma of vascularized corneas. Disruption of the Bowman membrane is associated with a significant increase of ANG2 stromal expression and proangiogenic macrophage infiltration in the corneal stroma [107]. Selective epithelial staining patterns for ANG2 are similar to those for sVEGFR-1, which is a potent soluble inhibitor of VEGF and angiogenesis [108]. Proangiogenic ANG2 is sequestered just above the basal epithelial membrane, and membrane disruption may be associated with ANG2 diffusion into the stroma. Interestingly, ANG2 stromal expression is associated with the massive infiltration of proangiogenic macrophages, whose numbers nearly tripled after Bowman removal [107].

Finally, platelet-derived growth factor (PDGF) is another late-phase vascular biomarker. PDGF has an angiogenic effect on sprouting endothelial cells, promotes capillary maturation by recruiting pericytes to growing vessels, and is necessary for pericyte viability [105,106].

## 5. Physical Properties of the Cornea as Biomarkers of Graft Survival

Central corneal thickness (CCT) is a widely utilized physical measurement of the cornea used for the accurate measurement of intraocular pressure (IOP) in glaucoma patients and for calculating the appropriate laser ablation that is needed for refractive surgeries to prevent resultant side effects, including corneal ectasia. These applications, however, are mainly concerned with the one-dimensional effects of thickness, such as variations in CCT causing artifacts in Goldmann applanation tonometer IOP measurements or the iatrogenic decrease in CCT seen in laser-assisted in situ keratomileusis (LASIK) procedures [113,114,115]. To explore the implications of CCT on corneal allograft placements, there have been numerous attempts to understand the physiology affecting CCT and the changes in CCT that can reveal underlying pathologies (Table 4).

Studies have revealed associations between CCT in patients with diabetes mellitus and hyperglycemia [118], speculating that the hyperglycemia-induced collagen disulfide crosslinks and osmotic fluid in the intracorneal spaces contribute to the shift in endothelial dynamics [119]. Further exploiting the corneal stromal permeability to fluids, CCT can also signify the level of hydration and activity of endothelial pumps that push fluids out of the stroma to prevent edematous buildup [120,121,122]. These pumps are altered in decompensated states, including Fuchs’ endothelial corneal dystrophy. Together with additional corneal physical properties, including hysteresis, resistance factor, and air pressure curves, the screening of subclinical presentations, such as forme fruste keratoconus, have become more reliable [127]. These findings suggest that corneal thickness reflects a wide range of underlying physiological mechanisms and this property can be applied to a multitude of other disease states, including corneal allograft rejection.

A report from the Corneal Donor Study, which searched for causes and associations of corneal allograft failure, posited that CCT has the potential to predict diagnoses of corneal edema, increases in IOP > 25 mmHg during the first postoperative month, and graft failure [116,117]. Interestingly, endothelial cell density (ECD), another measurement used for graft failure assessment, has shown little correlation with CCT. ECD only accounted for <10% of the variance in CCT, yet both parameters were independently predictive of graft failure. Although the authors caution against the use of CCT as a proxy for ECD, these results suggest that CCT may represent a novel and distinct niche in predicting graft failure and may justify further investigation on the mechanisms of its predictive strength. Currently, concomitant examination of corneal thickness kinetics and the use of specular endothelial micrography could expand the range of endothelial damage observed by clinicians, from the structural changes in the endothelium to the more extensive effects of inflammation and immune responses. Hence, the importance of deciphering the implications of a given CCT is increasing and is fueled by the rising concrete evidence that supports the highly genetic and inheritable component of CCT. The heritability of CCT seems to lie between 60% and 90%, suggesting that CCT is one of most highly heritable traits [128]. The connection between CCT and glaucoma has led to multiple genome-wide studies (GWS) conducted to determine loci that affect CCT. Lu et al. reported that there was a significant association between CCT variance and single-nucleotide polymorphisms (SNPs) near the ZNF469 gene (16q24), which is implicated in the development of Brittle Cornea Syndrome (BCS) [123]. BCS is a heritable disease characterized by extreme thinning of the cornea and skin and joint changes due to collagen dysfunction, thus providing a potential connection between the heritability of CCT and factors that affect corneal collagen formation. This finding aligns with the corneal thinning observed in certain connective tissue disorders that cause primary or secondary collagen malformation, including Ehlers–Danlos syndrome and osteogenesis imperfecta, further underscoring the importance of collagen in determining corneal architecture [129,130,131]. Not surprisingly, studies have reported that the COL5A1 gene, which also causes a variant of Ehlers–Danlos syndrome, and the COL8A2 gene are loci that both contribute to CCT variance [124,125]. COL8A2 mutations have been previously reported in patients with posterior polymorphous corneal dystrophy and Fuchs’ endothelial corneal dystrophy, both of which induce dystrophic changes to the cornea. Although these mutation hotspots are not directly linked to graft rejection, the connection between these loci and CCT may become valuable as the links between increased CCT and graft rejection are revealed.

Genome-wide association studies (GWAS) have also identified loci that are directly connected to corneal rejection, including certain minor histocompatibility antigens (mHag) loci, suggesting benefit to mHag matching in corneal allograft placements. Nicholls et al. conducted a GWAS on a swine model that underwent mismatched corneal transplantation and revealed four mHag loci that were associated with allograft rejection [126]. While three were novel findings, one block of SNPs spanned a region on chromosome 1 that contained the Zfp106 gene that encodes H-3a epitopes, and has previously been shown to mediate corneal graft rejection. Notably, a β-2M homologue was also located within the block of SNPs that has been shown to contribute to skin graft rejections [132]. Together with findings from the CCTS regarding the role of minor histocompatibility complex antigens in corneal graft rejection [133], genomic SNP analyses of corneal rejection-associated hotspots hold promise in prognosticating corneal allograft rejection. Furthermore, full genomic analysis of donors and recipients that examines individual corneal functional capabilities and immunologic characteristics could open up the possibility of precision gene therapy, such as individualized anti-angiogenic and anti-apoptotic gene therapy, to target rejection-promotive genes that vary in every patient [134].

## 6. Markers of Wound Healing

Neovessel formation is often a physiological process that is involved in tissue repair. Tissue damage is typically followed by an inflammatory response that protects the tissue from infection and promotes wound healing [135]. The innate arm of the immune system, particularly macrophages [136,137], facilitates wound healing by generating proangiogenic factors. Several studies have shown that corneal injury leads to the recruitment of VEGF-A-secreting macrophages and the induction of hemangiogenesis [138]. Blood vessels sprout at the edge of the injury and enable the formation of granulation tissue, which consists of fibroblasts and collagen [139]. Growth and maturation of blood vessels at the site of injury is usually a tightly controlled process and, once the tissue is repaired, the blood vessels regress [140]. Numerous physiological antiangiogenic factors, such as angiostatin, endostatin, tumstatin, vasohibin, fragments of prolactin, and growth hormones, facilitate this regression [141]. The regression of the vessels is then associated with the resolution of the inflammation [142]. Postgrafting wound healing involves the integrated actions of multiple growth factors, cytokines, and proteases that are produced by epithelial cells, stromal keratocytes, inflammatory cells, and lacrimal gland cells [143]. Table 5 lists the main markers of wound healing.

Multiple cytokines are released from the injured epithelium and epithelial basement membrane, including IL-1, TNF-alpha [144], bone morphogenic proteins (BMP) 2 and 4, epidermal growth factor (EGF), and PDGF [145]. These factors, along with others derived from the tears, trigger a variety of responses in underlying stromal keratocytes, including IL-1-mediated synthesis of Fas ligand. Keratocyte Fas ligand binds to the Fas receptor on nearby keratocytes and induces apoptosis [144] with minimal collateral damage from local cell lysis and liposomal enzyme release. In addition, it can induce autocrine suicide in keratocytes that already express Fas. A compromised epithelial barrier potentiates the effects of epithelial and lacrimal cytokines by providing direct access to the stroma.

Soon after wounding, extracellular matrix (ECM) proteins, such as fibronectin, fibrinogen/fibrin, laminin, and tenascin, are produced by both the basal cells and stromal keratocytes and appear on the denuded surface. Tumor growth factor (TGF)-β is known to enhance fibrosis and is thought to stimulate CTGF gene expression [146]. Studies have shown that corneal fibroblasts, keratocytes, and inflammatory cells may produce IL-1α and/or IL-1β that may act in a paracrine fashion to regulate myofibroblast apoptosis. Cao et al. assessed 1176 genes and found the expression of 37 of these genes are upregulated and the expression of 27 genes are downregulated more than 5-fold in the healing corneas when compared with those in the normal, uninjured corneas. IL-1, laminin-5, and thrombospondin-1 have all been found to be induced in the corneas in response to excimer laser treatment. The upregulated genes include intercellular adhesion molecule (ICAM)-1, macrophage inflammatory proteins, suppressors of cytokine signaling proteins (SOCS), IL-10 receptor, and galectin-7. The downregulated genes include connexin-31, a gap junction protein; ZO1 and occludin, tight junction proteins; and Smad2, a key component in the TGF signaling pathway [147].

MMPs also regulate corneal avascularity. MMP-7 (matrilysin) appears to protect against neovascularization, whereas MMP-14 is upregulated in the cornea in response to injury [143]. Studies have reported that prostaglandin E2 (PGE2) synthesis is increased in injured rabbit corneal endothelial cells [143,148]. Studies have also revealed that the concentration of EGF in tears changes rapidly following a corneal injury. The concentration of EGF is lower in unstimulated tears and higher in stimulated tears [149]. However, after chronic tearing, which occurs in patients with persistent epithelial defects or corneal ulcers, the concentration of EGF in tears decreases. This decrease is likely caused by the exhaustion of the lacrimal gland reserves of secreted proteins [150].

Other growth factors, including TGF-β, IL-1, acidic fibroblast growth factor (aFGF), bFGF, and PDGF, have been detected in tears, and PDGF concentrations were reported to increase in the tears of patients following surgery, suggesting their role in corneal wound healing [151]. Moreover, studies have reported that epiregulin is upregulated in limbal epithelial basal cells when compared with central cornea cells in mice. This upregulation of epiregulin increases corneal epithelial cell proliferation in vitro by activating EGFR and increasing the expressions of HB-EGF and Amphiregulin [152,153]. These findings suggest that epiregulin plays a role in the maintenance and proliferative capacity of limbal basal cells [154].

After corneal epithelial wounding, human growth factor (HGF) expression is upregulated in keratocytes [155] and epithelial cells [156,157], which may contribute to the epithelial wound healing process. While the rapid overexpression of insulin-like growth factor (IGF)-I in wounded mice cornea stimulates limbal cell differentiation with no effects on limbal cell proliferation [154,158], urokinase-type plasminogen activator (uPA/PLAU) is upregulated in wounded corneal epithelial cells and may stimulate cell migration [159].

Finally, damage triggers a release of inflammatory cytokines, mainly IL-1 (α and β), from epithelial cells and/or tears [143] that cause rapid apoptosis through Fas/Fas ligand system and necrosis of anterior keratocytes that induce corneal cell turnover.

**Table 5:** Markers of wound healing.

Markers of Wound Healing	Clinical Significance	References
IL-1α, IL-1β	Paracrine regulation of myofibroblasts apoptosis	[143,145,147]
TNF-α	Triggers stromal keratocytes responses, including IL-1-mediated synthesis of Fas ligand	[144,145]
EGF	Reflects level of intrastromal inflammation. Responds to key inflammatory mediators including IL-1 and TNF. Observed as early as 2 months before rejection. Levels decreased as immunosuppressant treatment progresses.	[145]
PDGF	Sub-basal and endothelial immune cell density increases associated with graft rejection. Reflects levels of stromal inflammation by responding to inflammatory mediators.	[145,151]
aFGF, bFGF	Binds to VEGF-A; VEGF-C; VEGF-C and D, respectively. Can act as anti-angiogenic factors in the corneal epithelial cells.	[151]
uPA	Corneal epithelial cells migration and proliferation	[159]

Abbreviation listed as followed. IL: Interleukin; TNF: Tumor necrosis factor; PDGF: platelet derived growth; EGF: epidermal growth factor; aFGF: acidic fibroblast growth factor; bFGF: basic fibroblast growth factor; uPA: urokinase-type plasminogen activator.

## 7. Future Directions

Various physical, cellular, and molecular biomarkers have been shown to influence the outcome of corneal grafts. However, it is important to identify the optimal biomarkers for prediction of graft outcomes. Omics analysis of host and corneal grafts has not been conducted and could reveal these key markers [160]. Extracting information from a large number of biomarkers is a challenge; however, well-trained clinicians may be able to glean diagnostic and prognostic information from a smaller number of markers. Mathematical technologies are being developed to estimate the patient state from a large set of biomarkers [161] or from a very short time-series of single biomarker [162,163]. Technologies like Bayesian belief networks and random survival forests constructed from conditional inference trees [162] have been recently developed to predict solid organ graft failure and can in principle be applied to corneal grafts [164]. Mathematical models can also help in understanding the mechanisms involved in corneal graft rejection and response to therapy [43]. These technologies may be used to predict graft survival and the progression of eye diseases in general.

Engineered corneas are emerging as promising alternatives to allografts. Advances in 3D bioprinting [165] and induced pluripotent stem cells (iPSC) technology [166] are enabling the construction of cornea mimics. Biomarkers that determine the survival of these engineered grafts still need to be identified and will expectedly depend on the engineering approach used. However, studies on allograft survival may guide the development of engineered grafts with improved survival.

## Figures and Tables

**Table 1:** Candidate biomarkers for detection and prediction of corneal allograft rejection.

Biomarkers	Clinical Significance	References
ABO Blood Group	Minor histocompatibility complex antigen mismatch implicated in allograft rejection.	[3,4]
HLA-DR	Major histocompatibility complex antigen mismatch implicated in allograft rejection for high-risk bed.	[5,6,7,8]
Activated Keratocytes	Reflect level of intrastromal inflammation. Respond to key inflammatory mediators including IL-1 and TNF-α. Observed as early as 2 months before rejection. Levels decrease as immunosuppressant treatment progresses.	[9,10,11,12,13,14,15]
Immune Cell Density	Sub-basal and endothelial immune cell density increase associated with graft rejection. Reflects levels of stromal inflammation by responding to inflammatory mediators.	[10,16]
Angio-/Lymphangiogenic Markers VEGFR-1, 2, 3	Binds to VEGF-A; VEGF-C; VEGF-D, respectively. Can act as anti-angiogenic factors in the corneal epithelial cells.	[17,18,19,20,21,22]
VEGF-A, C, D	Directly promotes corneal angio/lymphangiogenesis in the absence of above anti-angiogenic receptor.	
Inflammatory Markers IL-1, IL-6, IL-8, IL-17A, TNF-α	Proinflammatory cytokines upregulated post-transplantation.	[13,23,24,25,26,27]
MIP-1α, MIP-1β, MIP-2, RANTES, CCL2, CCL20, CCL21	Proinflammatory chemokines upregulated post-transplantation. Promote corneal acquisition of MHC class II cells and APC.	[21,24,28,29,30,31]
IL-2, IL-4, IL-5, IFN-γ	Protective factors (IL-2 and IL-5) and hazardous factors (IL-4 and IFN-γ) within the AqH. Candidate markers for prognosticating post-operative immune responses.	[27]
C3a	Complement pathway product. High levels in the AqH associated with graft rejection.	[32]
MHC class I-related chain A (MICA)	Expression induced by IFN-γ in corneal epithelial and endothelial cells. Connection to stimulation of CD8^+^ cells and subsequent promotion of immune response.	[33]
ICAM-1, VLA-1	Adhesion molecules targeted by immune cells. Expression upregulated in inflammatory states and promote acquisition of MHC class II cells and APC in the cornea.	[34,35,36,37]
Antigen Presenting Cells and Surface Proteins CD11c^+^(Dendritic cells)	Upregulation within 24 h of inflammation. Showed increased expression of MHC class II molecules in inflammatory states.	[38]
CD11c^−^/CD11b^+^ (Monocyte/Macrophage)	Migrates throughout the stroma (normally confined to posterior stroma) during inflammatory states.	[38]
CD80, CD86, CD40	Co-stimulatory molecules expressed on APCs, of which their expression is increased due to increased proinflammatory cytokines post-transplantation.	[7,38,39]
CCR7	Promotes CCL21-dependent APC migration to the cornea through afferent lymphatics.	[30]
T Cells and Surface Proteins Foxp3 (Treg)	Releases IL-10 and TGF. Correlated with reduced allograft rejection.	[16,40,41]
CD8^+^/IFN-γ^+^	High levels in the AqH associated with prognostication of allograft rejection.	[32]

Abbreviation listed as followed. HLA: human leukocyte antigen; VEGFR: vascular endothelial growth factor receptor; VEGF: vascular endothelial growth factor; IL: interleukin; TNF: tumor necrosis factor; MIP: macrophage inflammatory protein; RANTES: regulated on activation, normal T cell expressed and secreted; CCL: CC chemokine ligand; IFN: interferon; MHC: major histocompatibility complex; ICAM: intercellular adhesion molecule; VLA: very late antigen; CD: cluster of differentiation; Foxp3: forkhead boxprotein P3; APC: antigen-presenting cell; AqH: aqueous humor; TGF: tumor growth factor.

**Table 2:** Biomarkers for graft response related with corneal endothelial cells.

Biomarkers	Clinical Significance	References
Endothelial cell density (ECD, cell counts/mm^2^)	Lower ECD preoperatively and 2 months postoperatively was significantly correlated with the development of late endothelial failure after PKP. The lower ECD at 6 months postoperatively showed strong correlation with graft failure from endothelial decompensation.	[63,65] [66,67]
	Lower graft ECD was identified as a significant predisposing factor for lower postoperative ECD, but not for graft failure after DSAEK. Lower graft ECD was found as a significant risk factor for higher postoperative ECD loss by multinominal regression analysis after DMEK.	[70] [71]
Endothelial cell morphology Polymegethism (coefficient of variation of cell area, %)	Clinically valuable marker of the state of the endothelium	[15]
Pleomorphism (hexagonality, %) Spring constant *K* *	Valuable morphometric parameter of the state of the endothelium Lower hexagonality at 6 months after PKP showed a suggestive trend of higher graft failure. Positive correlation with CD166+/CD24–/CD105–/CD44– effector cell fraction for injection of cultured HCECs with a ROCK inhibitor. Preoperative *K* showed best classification accuracy with ECD at postoperative 6 months compared with other parameters, including effector cell fraction, preoperative ECD, and preoperative hexagonality.	[15] [67] [61]
Genes ANAPC1	A cell cycle-regulated E3 ubiquitin ligase which controls progression through mitosis and the G1 phase of the cell cycle. An intergenic variant (rs78658973[A]) close to ANAPC1 was found to have a strong association with decreased ECD.	[72]

* the collective order of HCECs, calculated by the second derivative of the function summated for the number of neighbor cells according to distance from each reference cell. Abbreviation listed as followed. ECD: endothelial cell density, PKP: penetrating keratoplasty, DSAEK: Descemet’s stripping automated endothelial keratoplasty, DMEK: Descemet’s membrane endothelial keratoplasty, HCES: human corneal endothelial cell, ROCK: rho-associated protein kinase, ANAPC1: anaphase-promoting complex subunit 1.

**Table 4:** Clinical factors and gene loci associated with CCT.

Associated Factors	Clinical Significance	References
Graft Failure	CCT was associated with graft failure independent of the prediction made through ECD. The possibility of an unknown mechanism connecting CCT to graft failure has been posited.	[116,117]
Diabetes and Hyperglycemia	Associated with corneal endothelial dysfunction and resultant stromal hydration of the cornea. Osmotic fluid shifts and collagen cross-linkage are likely etiologies.	[118,119]
Endothelial Decompensation, Corneal Edema	Diseases involving endothelial dysfunction, such as Fuchs’ endothelial corneal dystrophy, progress into corneal edema. Resultant increase in CCT is a reliable method to measure disease progression.	[120,121,122]
ΔIOP > 25 mmHg (post-operation)	CCT was predictive of IOP increase 1 month postoperatively. Preoperative glaucoma was associated with early graft failure. CCT may represent the underlying physiologic link that connects glaucoma and graft failure.	[116,117]
GenesZNF469	Possible regulator of collagen synthesis and/or organization. Implicated in the development of Brittle Cornea syndrome, which exhibits markedly reduced CCT.	[123]
COL5A1	Encodes for the alpha-1 chain of type V collagen. Associated with a variation of Ehlers–Danlos syndrome, which also exhibits reduced CCT.	[124,125]
COL8A2	Encodes for the alpha-2 chain of type VIII collagen. Associated with posterior polymorphous corneal dystrophy and Fuchs’ endothelial corneal dystrophy, characterized by changes in the endothelial layer and Descemet’s membrane.	[124,125]
ZFP106	Contains an mHag loci which encodes for H-3a epitopes. These loci were previously shown to mediate corneal graft allograft rejection.	[126]

Abbreviation listed as followed. CCT: central corneal thickness; ECD: endothelial cell density; IOP: intraocular pressure; ZNF: zinc finger; ZFP: zinc finger protein; COL: collagen; mHag: minor histocompatibility antigen.
